# The Significant Therapeutic Effects of Chinese Scorpion: Modern Scientific Exploration of Ion Channels

**DOI:** 10.3390/ph17121735

**Published:** 2024-12-22

**Authors:** Yueyuan Zheng, Qiuyi Wen, Yushi Huang, Dean Guo

**Affiliations:** 1 School of Pharmacy, Guangdong Pharmaceutical University, Guangzhou 510006, China; zhengyueyuan@zidd.ac.cn (Y.Z.); 13640465830@163.com (Q.W.); h13198194801@163.com (Y.H.); 2 Zhongshan Institute for Drug Discovery, Shanghai Institute of Materia Medica, Chinese Academy of Sciences, Zhongshan 528400, China

**Keywords:** *Buthus martensii* Karsch, ion channel, chemical components, pharmacological effects

## Abstract

Chinese scorpion (CS), a traditional animal-based medicine used for over a millennium, has been documented since AD 935–960. It is derived from the scorpion *Buthus martensii* Karsch and is used to treat various ailments such as stroke, epilepsy, rheumatism, and more. Modern research has identified the pharmacological mechanisms behind its traditional uses, with active components like venom and proteins showing analgesic, antitumor, antiepileptic, and antithrombotic effects. Studies reveal that CS affects ion channels, crucial for cellular functions, through interactions with sodium, potassium, and calcium channels, potentially explaining its therapeutic effects. Future research aims to elucidate the precise mechanisms, target specific ion channel subtypes, and validate clinical efficacy and safety, paving the way for novel therapies based on these natural compounds.

## 1. Introduction

Chinese scorpion (CS), also referred to as Quanxie or Scorpio, is a traditional animal-based medicine that has been utilized for over a millennium according to many ancient Chinese medical books. It comprises the dry body of the scorpion *Buthus martensii* Karsch (BmK). The earliest documentation of CS dates back to the Shu Ben Cao (AD 935–960). Subsequent texts, such as the Kai Bao Ben Cao (AD 973–974), Ben Cao Yan Yi (AD 1116), Ben Cao Gang Mu (AD 1552–1578), and Ben Cao Bei Yao (AD 1681–1694), further document the functional indications of scorpions. In addition, 1349 prescriptions of scorpions have been included in 167 ancient Chinese works of medical literature. These indicate the efficacy of scorpions in treating stroke, epilepsy, rheumatism, hernia, chancre, and tetanus, etc. [1,2,3,4,5,6]. As of the current edition of the Chinese Pharmacopoeia (2020), there are 34 types of CS preparations, such as the *Naoxintong* capsule, *Dianxianping* tablet, and *Huoxue Zhuangjin* pill. Notably, the *Huoxue Zhuangjin* pill is primarily used to treat rheumatism, spasms, convulsions, hemiplegia, palpitations, cerebral infarction, coronary heart disease, angina pectoris, and other diseases [7].

In recent years, modern scientific research has begun to uncover the pharmacological mechanisms behind the traditional uses of CS. Studies have shown that the active components in CS, such as scorpion venom and proteins, exhibit analgesic, antitumor, antiepileptic, and antithrombotic effects. For example, one study found that BmK IT2, a peptide isolated from CS, significantly reduced inflammation and pain in rats with complete Freund’s adjuvant-induced arthritis [8]. Another study demonstrated that CS extract inhibited the proliferation of tumor cells and induced apoptosis in vitro, suggesting its potential as an anticancer agent [9]. Moreover, steroids, lecithin, trimethylamine, betaine, organic acids, and other small-molecule compounds isolated from CS also form the material basis for its efficacy [10].

One area of intense research is the effect of CS on ion channels, which are protein molecules that form holes in the cell membrane, allowing ions to pass in and out of the cell [11]. These channels play a crucial role in maintaining electrical potential differentiation across the membrane, which is essential for cellular functions like muscle contraction and nerve signaling. However, any disturbance of ion channels can result in channelopathies, which can lead to various diseases such as cardiovascular, immunological, neurological, and even cancer. Scorpion venom, particularly its peptides, interacts with various types of ion channels, leading to their activation or inhibition, and as such, has utility for the development of drugs with targeted specificity [12,13,14,15,16]. For example, peptides like BmK IT2 modulate the sodium channels involved in generating action potentials, influencing pain signaling pathways and offering a potential mechanism for their analgesic effects [8]. In antitumor research, CS extract inhibits tumor cell proliferation and induces apoptosis, possibly by interacting with the potassium channels often overexpressed in cancer cells [9]. This could disrupt the electrochemical balance necessary for tumor cell survival.

The anti-inflammatory effects of CS may also result from its action on ion channels, such as calcium channels, which play a role in the inflammatory process by regulating calcium ions [17]. Scorpion venom compounds could suppress inflammation by modulating intracellular calcium levels. Additionally, small-molecule compounds from CS, like steroids and organic acids, may contribute to its pharmacological effects by interacting with ion channels or other cellular targets, potentially synergizing with venom peptides to enhance efficacy or provide additional benefits [18].

Although scorpion toxin encompasses a myriad of toxic polypeptides with varied functionalities, as meticulously reviewed by Ahmadi et al. [19], this review is explicitly focused on exploring the impact of CS on ion channels. This exploration elucidates its diverse pharmacological activities, thereby validating its traditional medicinal uses and underscoring its potential as a novel source for therapeutic agents. Future research should focus on the precise mechanisms of action, target ion channel subtypes, and clinical trial efficacy and safety. This could validate traditional uses and lead to new therapies based on these natural compounds.

## 2. Chemical Components Affecting Ion Channels

**Figure 1:**
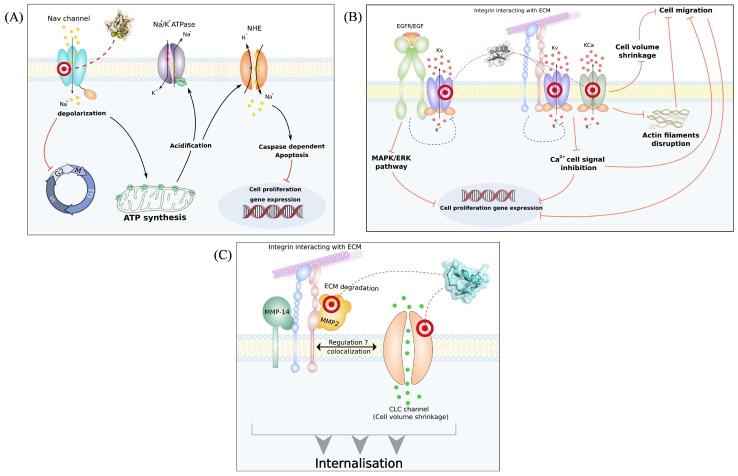
The interaction of scorpion toxins with specific ion channels elicits antitumor effects. (**A**) Modulation of cellular targets involved in cancer cell proliferation and apoptosis mechanisms by sodium channels scorpion toxins. (**B**) Inhibition of cellular targets involved in cancer cell proliferation and migration mechanisms by potassium channels scorpion toxins. (**C**) Inhibition of cellular targets involved in cancer cell invasion and migration mechanisms by chloride channel scorpion toxins [12] (reproduced with permission from Najet Srairi-Abid, Cell Calcium; published by Elsevier, 2019).

Scorpion venom toxins are small peptides composed of 20–80 amino acids, containing 3–4 pairs of disulfide bonds, with molecular weights ranging from 6000 to 9000 Da. These peptides exhibit high specificity and unique biological activities. Based on the bond connections, they can be classified into disulfide-bridged peptides (DBPs) and non-disulfide-bridged peptides (NDBPs) [20,21]. Further classification based on the types of ion channels that DBPs interact with Na^+^, K^+^, Cl^−^, and Ca^2+^ channel scorpion toxins, each exhibiting different pharmacological effects, such as antitumor (Figure 1), analgesic, antiepileptic, and antibacterial activities [22]. Studies have indicated that these interactions are related to the structural differences of scorpion venom toxins. Long-chain scorpion toxins specifically target Na^+^ channels, while short-chain scorpion toxins primarily affect K^+^, Cl^−^, and Ca^2+^ channels [2]. The following section introduces the different types of scorpion venom toxins found in CS.

### 2.1. Scorpion Toxins Targeting Sodium Channels

Voltage-gated sodium channels (VGSCs) consist of *α*-subunit and *β*-subunits. The *α*-subunit contains four highly homologous domains (I, II, III, IV), each with six *α*-helical transmembrane segments (S1–S6). The *β*-subunits assist in regulating the function of the *α*-subunit. VGSCs can be classified into nine subtypes (Nav1.1–Nav1.9) based on the structure of the *α*-subunit. Among these, Nav1.3, Nav1.7, Nav1.8, and Nav1.9 have been identified as potential targets for analgesic drugs [23,24,25].

Scorpion venom toxins that target sodium channels are primarily classified into *α*-scorpion toxins and *β*-scorpion toxins, distinguished by their binding sites and electrophysiological characteristics. *α*-scorpion toxins are further subdivided into classic *α*-scorpion toxins, insect *α*-scorpion toxins, and *α*-like scorpion toxins, depending on their specificity toward mammals and insects. These toxins can bind to site 3 of VGSCs, thereby inhibiting channel inactivation at the cell membrane level. Similarly, *β*-scorpion toxins are categorized into classic *β*-scorpion toxins, insect *β*-scorpion toxins, and *β*-like scorpion toxins. Insect *β*-scorpion toxins are further divided into excitatory and inhibitory insect toxins [26]. These *β*-scorpion toxins bind to site 4 of VGSCs and modulate their activation. In all scorpion venom toxins, excitatory insect toxins can enhance peak sodium currents and maintain the continuous opening of sodium channels. In contrast, inhibitory insect toxins can suppress the occurrence of action potentials and inhibit sodium currents [19,26].

#### 2.1.1. α-Scorpion Toxin

*α*-scorpion toxins bind to sodium channels in a voltage-dependent manner, with changes in membrane potential affecting the structure of receptor sites. This interaction activates the sodium channels, thereby slowing their inactivation process [26]. For instance, the *α*-like scorpion toxin BmK I can induce pain by directly modulating Nav1.8 currents and inhibiting the inactivation of Nav1.8. This results in the prolonged opening of sodium channels and sustained influx of sodium ions [27]. Consequently, BmK I reduces the threshold for neuronal excitability and enhances the frequency of action potentials. This leads to overexcitement of dorsal root ganglia (DRG) neurons, which can display pain behaviors in rats [27].

#### 2.1.2. *β*-Scorpion Toxin

*β*-scorpion toxins modify the voltage dependence of voltage-gated sodium channels (VGSCs) by binding to receptor site 4. This interaction leads to the subthreshold opening of ion channels and a consequent reduction in peak current amplitude [28].

BmK AS, a *β*-like scorpion neurotoxin, specifically modulates site 4 of voltage-gated sodium channels (VGSCs) by inhibiting sodium currents in a dose-dependent manner, exhibiting anticonvulsant properties in vivo. A study investigated the impact of BmK AS on acute seizures in unanesthetized rats induced by pentylenetetrazole (PTZ) and pilocarpine [29]. The results demonstrated that BmK AS significantly suppressed the peak sodium current in cultured hippocampal neurons by 84% and inhibited PTZ-induced seizures. However, it had only a slight effect on pilocarpine-induced epileptic seizures, which are not associated with the peak sodium current. Other studies have shown that BmK AS stabilizes both the closed and open states of the Nav1.3 channel, promoting steady activation and inhibiting slow inactivation, thereby exhibiting anti-inflammatory, analgesic, and anticonvulsant activities [30].

**Figure 2:**
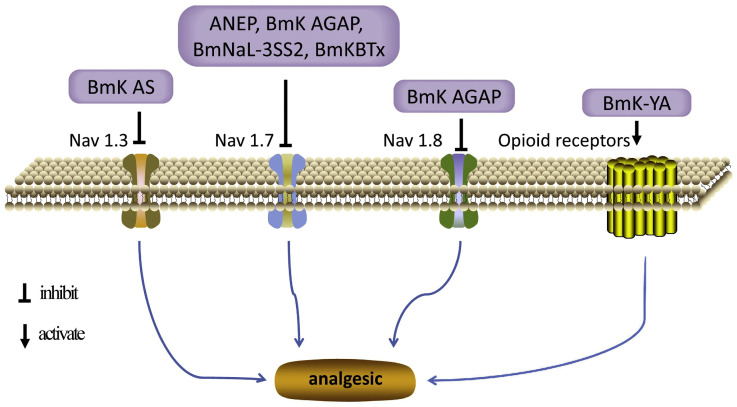
Mode of action for peptides with analgesic activity [22] (reproduced with permission from Zhongjie Li, Peptides; published by Elsevier, 2019).

Another *β*-scorpion toxin from CS, the antineuroexcitation peptide (ANEP), has shown analgesic activity in a mouse writhing test and is associated with the Nav1.7 channel (Figure 2) [31,32]. Nav1.7 is predominantly distributed in sympathetic neurons, the olfactory epithelium, and dorsal root ganglion (DRG) neurons, playing a crucial role in the development of the acute and chronic peripheral pain associated with tissue and nerve injury. Molecular dynamics simulations, following homology modeling, molecular mechanics, and molecular dynamics in a biomembrane environment, revealed that ANEP predominantly interacts with the resting site 4 via amino acid residues located in both the *β*_2_-*β*_3_ loop and the ‘NC’ domain, leading to sodium channel activation. Under the activated state, the ‘pharmacophore’ and N-groove of ANEP interact with the *S*_3_-*S*_4_ loop of Nav1.7, enhancing channel activation in response to subsequent depolarization. This interaction results in a negative shift in activated voltage dependence [33].

**Figure 3:**
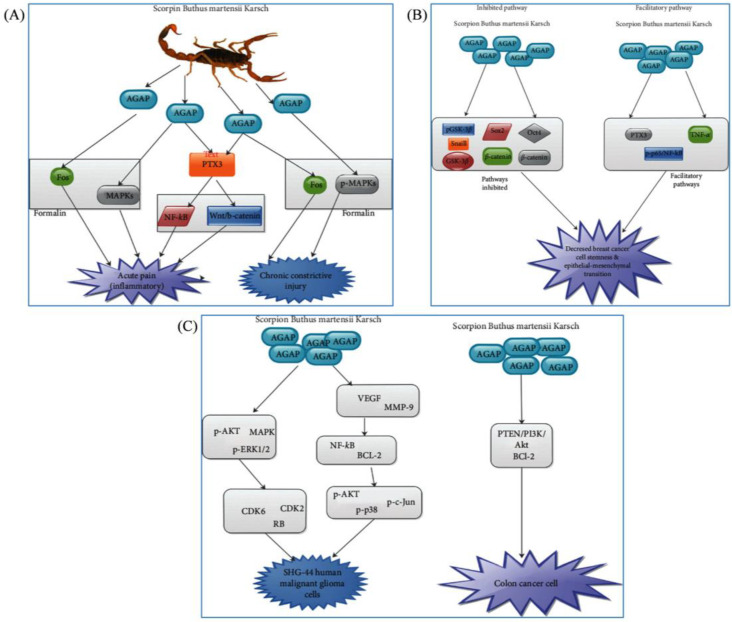
Diagram illustrating the pathways associated with AGAP. (**A**) AGAP induces analgesia by effectively alleviating acute inflammatory pain and chronic constrictive injury through the modulation of MAPK and Fos signaling pathways in formalin-induced models. (**B**) AGAP decreases breast cancer cell stemness and epithelial–mesenchymal transition. (**C**) AGAP influences SHG-44 human malignant glioma cells and colon cancer cells [24] (reproduced with permission from Seidu A. Richard, Evidence-Based Complementary and Alternative Medicine; published by Hindawi, 2020).

The analgesic-antitumor peptide (AGAP), derived from BmK, functions as a specific inhibitor of voltage-gated sodium channels by suppressing their mRNA transcription (Figure 3) [24]. Although AGAP can be classified as an *α*-scorpion toxin by structural analysis, electrophysiological studies reveal that AGAP may be defined as a *β*-scorpion toxin [34]. Studies have shown that AGAP reduces HVA calcium channels, specifically N-type calcium currents [35]. It targets the Nav1.7, Nav1.8, and Nav1.9 subtypes, thereby diminishing neuronal excitability and exerting analgesic effects [34,35]. Li et al. corroborated these findings, showing that 1 mM AGAP significantly reduced the currents of Nav1.8 by 59.4 ± 5.1% and Nav1.9 by 33.7 ± 6.6%, thereby confirming its pain-relieving properties via channel inhibition in small-diameter DRG neurons [35]. Xu et al. further elucidated that AGAP robustly impairs both hNav1.7 and hNav1.8, underscoring the involvement of these isoforms in its analgesic mechanism [34]. Nevertheless, the precise mechanisms underlying the analgesic impacts of AGAP on voltage-gated sodium channels (Navs) and its structural–functional correlation remain ambiguous. Furthermore, research indicates that recombinant AGAP (rAGAP) possesses anticancer properties, inhibiting the growth of malignancies including colorectal carcinoma, lymphoma, and glioma [36,37,38]. Its signaling pathway involves p27, Bcl-2, and Bax, but whether VGSCs are also implicated in this process remains an interesting question.

### 2.2. Scorpion Toxins Targeting Potassium Channels

**Figure 4:**
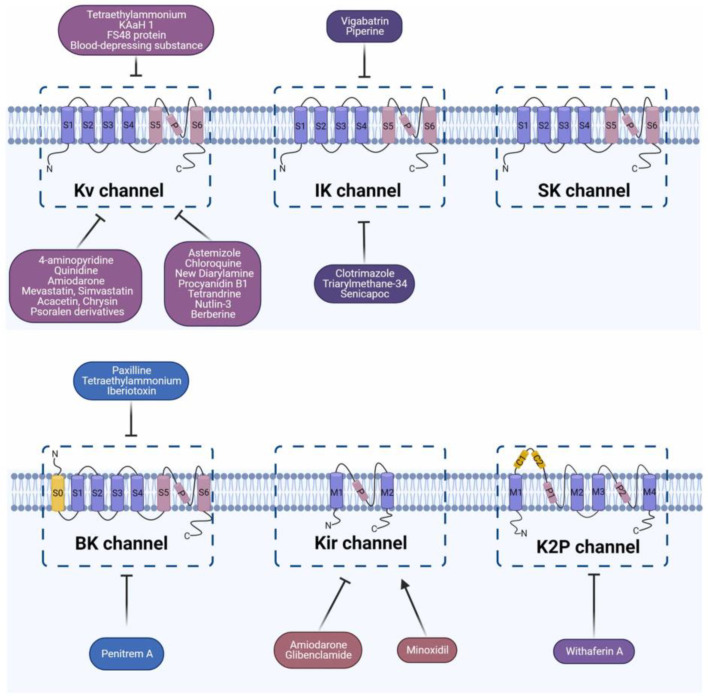
The structure of potassium channels [40] (reproduced with permission from Chenglai Xia, Biomedicine & Pharmacotherapy; published by Elsevier, 2023).

Potassium ion channels constitute the most numerous and complex family of ion channels. Recent studies have demonstrated their critical role in maintaining cellular ion homeostasis, ion transport, signal transduction, and other physiological activities (Figure 4) [39,40,41]. These channels can be classified into four major types: voltage-gated potassium channels (Kv), calcium-activated potassium channels (KCa), inwardly rectifying potassium channels (Kir), and tandem-pore-domain potassium channels (K2p) [42]. The voltage-gated potassium channel Kv family contains 12 channels (Kv1–Kv12) along with various subtypes [43].

Potassium channel toxins (KTxs) primarily act as inhibitors of potassium ion channels. Based on their sequences and cysteine pairs, KTxs are categorized into *α*, *β*, *γ*, *κ*, *δ*, *λ*, and *ε*-KTx subfamilies [19]. BmKTX is an *α*-KTx toxin purified from BmK venom, specifically inhibiting Kv1.3 potassium channels. In BmKTX, Arg23 serves as a new pore-blocking residue, mainly using the turn motif between the *α*-helix and antiparallel *β*-sheet structural domains as the channel-interacting interface to block the Kv1.3 channel [44]. BmKTX-D33H, the structural analog of BmKTX, employs conserved antiparallel *β*-sheet structural domains as the interacting interface, with its Gly11 residue interacting with the turret domain of the Kv1.3 channel. This analog can inhibit cytokine production and proliferation in Jurkat cells and human T cells. In rat models, BmKTX-D33H significantly improved T-cell-mediated delayed-type hypersensitivity (DTH) [45]. Thus, BmKTX and its analog BmKTX-D33H demonstrate significant potential in modulating immune responses and treating T-cell-mediated hypersensitivity reactions through their specific interactions with Kv1.3 potassium channels.

### 2.3. Scorpion Toxins Targeting Chloride Channels

Chloride ion channels exhibit various cellular functions, including osmotic regulation, cell migration, and cell proliferation. There are abundant chloride ion currents on the surface of glioma cells, and the movement of chloride ions influences cell volume changes in glioma cells, with the chloride channel 2 (ClC-2) and chloride channel 3 (ClC-3) superfamily being predominantly responsible for mediating. These channels are scarce in healthy tissues. Therefore, the expression of ClC-2 and ClC-3 channels highlights their association with glioma cell proliferation and invasiveness [46,47]. Scorpion venom, by specifically inhibiting these chloride channels, holds promise as a therapeutic strategy against glioma, acting through the modulation of chloride ion transport and consequent cellular processes.

#### 2.3.1. Chlorotoxin

Chlorotoxin (CTX) is the first identified chloride channel inhibitor from scorpion toxins, isolated from the venom of *Leiurus quinquestriatus*. When CTX binds to the intracellular surface of ClC-3 channels, it blocks the low-conductance Cl^−^ channels, inhibiting chloride ion flow. This leads to a reduction in glioma cell volume and delays tumor cell migration and invasion [12]. Additionally, CTX disrupts ClC-3 by interacting with a complex protein composed of matrix metalloproteinase-2 (MMP-2), membrane type 1 matrix metalloprotease (MT1-MMP), and the tissue inhibitor of matrix metalloproteinase-2 (TIMP-2) on the surface of glioma cell membranes. MMP-2 is also a target of chloride channel scorpion peptides. It is expressed in gliomas and other tumors but is not present in normal brain tissue, where its presence is associated with the enzyme degradation of the extracellular matrix (ECM). CTX decreases the expression of MMP-2 on the cell surface through endocytosis and inhibits its enzymatic activity, thereby exhibiting anti-invasion effects on glioma cells [47,48].

#### 2.3.2. BmK CT

The short-chain neurotoxin BmK CT is the first chloride toxin-like peptide isolated from BmK, exhibiting a 68% amino acid sequence homology to CTX and can specifically inhibit chloride ion channels [22]. A study of rBmK CTa, expressed in a soluble form in *E. coli* BL21 (DE3), significantly inhibited the growth of the host bacterial strain, suggesting that BmK CT might specifically inhibit the chloride ion channel of prokaryotic organisms [49]. BmK CT and its radiolabeled derivatives, ^125^I-BmK CT and ^131^I-BmK CT, inhibited the expression of MMP-2, subsequently diminishing the invasiveness of glioma cells [50,51]. Further research indicates that BmK CT downregulates MMP-2 and MMP-9 expression by upregulating TIMP-2, which regulates matrix metalloproteinases (MMPs) [51]. Additionally, BmK CT enhances the sensitivity of apoptosis in U251 cells induced by TMZ through the downregulation of phosphorylated AKT (p-AKT) levels, thereby significantly inhibiting U251 cell proliferation and inducing apoptosis [52].

In conclusion, chlorotoxin and BmK CT have shown significant potential in inhibiting chloride ion channels and modulating associated cellular processes, offering promising therapeutic benefits for improving the efficacy of glioma treatments.

### 2.4. Scorpion Toxins Targeting Calcium Channels

Calcium, a divalent ion, was selected early in evolution as a signaling molecule utilized by both prokaryotes and eukaryotes [53]. Consequently, calcium channels are crucial for facilitating the transport of calcium ions across cell membranes, thereby supporting diverse physiological functions [54]. Ryanodine receptors (RyRs) are tetrameric calcium ion channels with a molecular weight of 2.26 MDa located on the intracellular membrane of the sarcoplasmic reticulum (SR). These receptors contain multiple domains that regulate calcium release and play a crucial role in muscle contraction and signal transmission. RyRs can be classified into three isoforms: RyR1 is predominantly found in skeletal muscle, where specific mutations in its transmembrane region can cause malignant hyperthermia (MH) and central core disease (CCD); RyR2 is abundant in cardiac muscle cells, and mutations in its amino-terminal, carboxy-terminal, and central domains are associated with catecholaminergic polymorphic ventricular tachycardia (CPVT); while RyR3 is less abundant and was originally isolated in the brain [55,56,57].

Scorpion venom peptides, such as Imperatoxin A (IpTxa) and Hadrucalcin (HdCa), exhibit therapeutic potential for various diseases by targeting specific ion channels. IpTxa, the first scorpion toxin identified with the capacity to bind to calcium ion channels, was isolated from the venom of the African scorpion *Pandinus imperator*. Similarly, HdCa is a basic 35 residue peptide purified from the venom of the Mexican scorpion *Hadrurus gertschi* [58,59]. They can specifically bind to RyRs and activate them, leading to the release of calcium ions from the SR into the cytoplasm of muscle cells, increasing intracellular calcium concentration, thereby affecting cardiac contraction [60].

### 2.5. Chemical Components Targeting Multiple Ion Channels

#### 2.5.1. Targeting Calcium-Activated Potassium Channels

**Figure 5:**
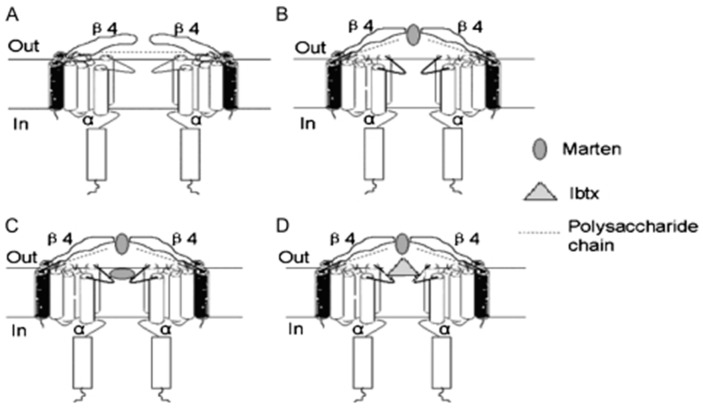
Proposed mechanism for the interaction between BK channels (*α* + *β*4) and martentoxin. The conformation of a normal open state (**A**) was changed by the application of martentoxin when the first steady complex was formed (**B**). Then, the low-affinity site was exposed to associate with martentoxin (**C**) or iberiotoxin (**D**) [62] (reproduced with permission from Yonghua Ji, Biophysical Journal; published by Elsevier, 2008).

Large-conductance calcium-activated potassium (BK) channels are one type of potassium ion channel, consisting of *α* subunit tetramers encoded by the single gene Slowpoke (Slo). In mammals, BK channels are categorized into four subtypes, each associated with different *β* subunits. Martentoxin, an *α*-KTx toxin comprising 37 amino acids, specifically inhibits BK channels. Its secondary structure features an *α*-helix connected to a triple-stranded *β*-sheet via three disulfide bridges [61]. Notably, martentoxin exhibits selectivity among different BK channel subtypes. For instance, it potently blocks BK channels (*α* + *β*4) but has less impact on those with one *α* subunit (Figure 5) [62]. The regulatory effect of martentoxin on BK channels is modulated by calcium concentration, with the toxin decreasing channel current at low calcium concentrations and increasing it at high calcium concentrations [61].

**Figure 6:**
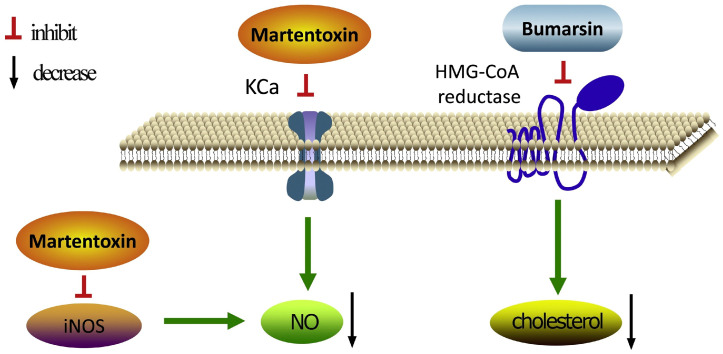
Mode of action of martentoxin and bumarsin. KCa: Ca^2+^-activated K^+^ channels; iNOS: inducible nitric oxide synthase; NO: nitric oxide [22] (reproduced with permission from Zhongjie Li, Peptides; published by Elsevier, 2019).

Yang et al. identified Phe1, Lys28, and Arg35 as critical amino acid residues for martentoxin’s interaction with BK channels through protein–protein docking, molecular dynamics simulations, and virtual alanine scanning [63]. In endothelial cells, the BK channel-mediated changes in membrane potential can influence calcium influx and nitric oxide (NO) release. NO acts as a regulator of vascular inflammation. Martentoxin inhibits the activity of inducible NOS (iNOS), which is mediated by TNF-α, thereby reducing NO production while enhancing endothelial NOS (eNOS) expression. Consequently, martentoxin holds potential therapeutic applications for treating vascular diseases (Figure 6) [64].

Additionally, LQ-VIII and LQ-X/2 were isolated from the scorpion venom of *Leiurus quinquestriatus hebraeus*. LQ-VIII can specifically block BK channels in guinea pig hepatocytes, while LQ-X/2 is the first toxin shown to block erythrocyte BK channels with high affinity [65].

#### 2.5.2. Modulating Calcium-Activated Chlorine Channels

Calcium-activated chloride channels (CaCCs) are present in almost every mammalian tissue. The molecular basis of CaCCs is anoctamin-1 (ANO1), also known as transmembrane protein 16A (TMEM16A) [66,67]. In smooth muscle tissues, CaCCs serve as the major anionic channels. The activation of CaCCs leads to chloride ion efflux and the depolarization of muscle cell membranes, thereby affecting the contraction and relaxation of smooth muscles [68]. The Lqh 7-1 peptide isolated from *Leiurus quinquestriatus hebraeus* scorpion venom can specifically inhibit CaCCs in rat portal vein myocytes, and the synthesized Lqh 7-1 peptide has the same affinity and inhibition potency as the natural toxin [69].

#### 2.5.3. Affecting on Na^+^-K^+^-ATPase

**Figure 7:**
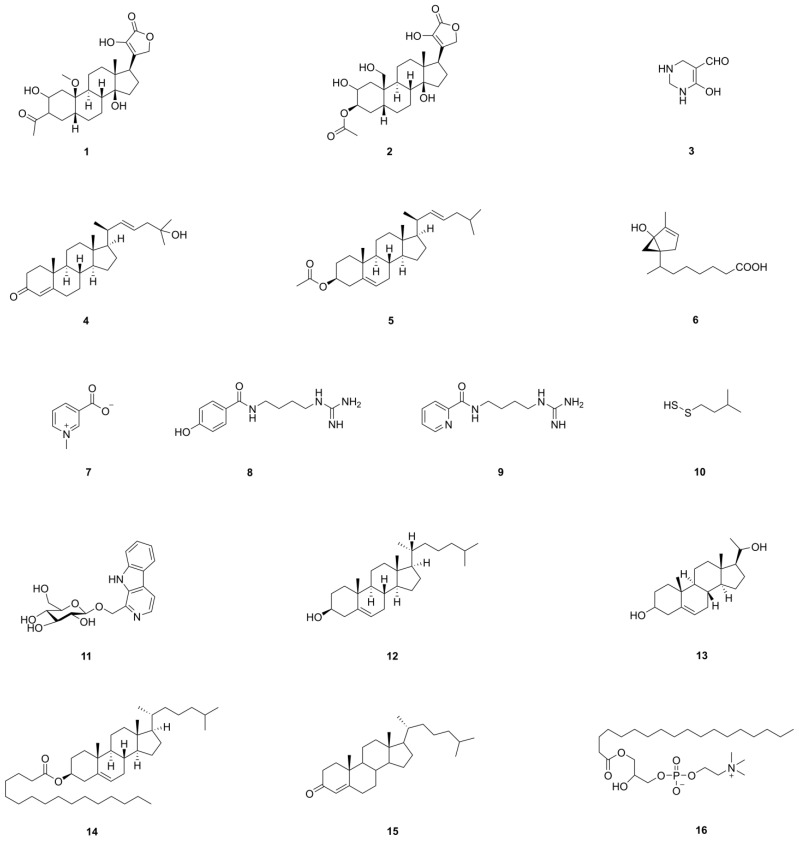
Structures of special small-molecule compounds in CS.

Cardiac glycosides, with steroids at their core and an unsaturated lactone ring attached to C-17, are found in plant families like Apocynaceae, Scrophulariaceae, Brassicaceae, and Liliaceae and the derivatives from toad venom [70,71]. They inhibit Na^+^-K^+^-ATPase, increasing intracellular Na^+^ and then increasing Ca^2+^ concentrations via a Na^+^/Ca^2+^ exchange mechanism (NCX), which enhances myocardial contractility [72,73]. Additionally, they affect Na^+^-K^+^-ATPase subunits and the balance of Na^+^, K^+^, and Ca^2+^ ions in tumor cells, disrupting signal transduction and inducing apoptosis [74,75]. Other antitumor mechanisms include HIF-1 inhibition, FGF-2 and NF-B suppression, topoisomerase inhibition, and cell cycle arrest [75,76]. Two cardiac glycoside compounds from CS show antibacterial activity (compound **1** and **2** of Table 1 and Figure 7), but their effects on Na^+^-K^+^-ATPase and antitumor activities need further study [77,78].

### 2.6. Other Components

#### 2.6.1. NDBPs

NDBPs are composed of 13–56 amino acid residues and possess a diverse range of sequences and flexible structures [79]. One characteristic of NDBPs is their irregular random helical structure in aqueous solutions, which transitions to an amphiphilic *α*-helical structure in 50–60% aqueous trifluoroethanol (TFE). Positively charged NDBPs can interact with the negatively charged phospholipid head group of the target cell lipid membrane through electrostatic forces. This interaction drives the formation of amphiphilic helices and the insertion of hydrophobic residues, thereby increasing activity. Through this mechanism, NDBPs can interact with a wide range of biological targets, significantly differing from the mechanism through which toxins target specific ion channels in specific biological targets [20,79,80].

BmKn2 and BmKb1 are NDBPs isolated from BmK, both of which are basic *α*-helical peptides with an amidated C-terminus. BmKn2 exhibits strong antibacterial activity against both Gram-positive and Gram-negative bacteria, whereas BmKb1 shows a weaker antibacterial activity [81]. Additionally, BmKn2 possesses antitumor activity, inducing apoptosis in human oral squamous cell carcinoma cells (HSC-4). It promotes the expression of proapoptotic genes such as caspase-3, -7, and -9; upregulates BAX; and decreases the expression of antiapoptotic BCL-2 [82,83]. BmKbpp is a 47 amino acid peptide that not only exhibits strong antibacterial activity against both Gram-positive and Gram-negative bacteria but also increases bradykinin activity [84].

#### 2.6.2. Cholesterol and Its Derivatives

Cholesterol is a critical source of bioactive substances, along with phospholipids that form the fundamental structure of cell membranes and participate in the biochemical activities of cell membranes [85]. Lv et al. found novel 5,22*E*-cholestadienol derivatives from CS. These compounds, specifically (–)-7*S*,8*S*,9*R*,13*R*,14*S*,17*R*,20*S*(22*E*)-3-oxocholesta-4,22(23)-dien-25-ol and (–)-7*S*,8*S*,9*R*,13*R*,14*S*,17*R*,20*S*-(22*E*)-3*b*-acetate,5(6)-22*E*-(23)-cholestadien (compound **4** and **5** of Table 1 and Figure 7), exhibit potent broad-spectrum antibacterial activity against *Bacillus subtilis*, *Staphylococcus aureus*, and *Pseudomonas aeruginosa* [86]. They interact with the target receptor proteins 2XRL or 1Q23 from bacterial ribosome unit A through specific bonding forces, thereby inhibiting protein synthesis and bacterial proliferation [86].

#### 2.6.3. Alkaloids

Liu et al. isolated two guanidine-type alkaloids from the 85% methanol extract of CS, confirming their structures as *N*-(4-guanidinobutyl)-4-hydroxybenzamide and *N*-(4-guanidinobutyl)picolinamide, named as Buthutin A and Buthutin B (compound **8** and **9** of Table 1 and Figure 7) [87]. Notably, Buthutin A exhibits a potent inhibitory activity against both acetylcholinesterase and butyrylcholinesterase. Buthutin A also demonstrates significant biometal-binding capabilities with Cu^2+^, Fe^2+^, Zn^2+^, and Al^3+^ ions, potentially mitigating oxidative damage induced by free radicals. This discovery provides possibilities for exploring therapeutic strategies against Alzheimer’s disease [87].

Kim isolated a novel *b*-carboline glucoalkaloid from the ethanol extract of BmK and determined it as harmanyl *b*-*D*-glucopyranoside, which inhibited *α*-glucosidase (compound **11** of Table 1 and Figure 7) [88].

#### 2.6.4. Small-Molecule Compounds

In-depth investigations into its chemical composition have revealed the presence of buthotoxin, steroidal derivatives, alkaloids, aliphatic acids, and amino acids [2,3]. The information and structures of these unique small-molecule compounds are presented in Table 1 and Figure 7. The specific mechanisms of these warrant further exploration. They may exert their pharmacological activities by interacting with specific enzymes, receptors, or ion channels to regulate cellular signaling or gene expression.

**Table 1:** Special small-molecule compounds in CS.

No.	Compound	Molecular Formula	Pharmacological Activity	Ref.
**1**	2*b*-,22-dihydroxy,3-acetoxyl,20-methoxy-cardenolidol	C_28_H_45_O_7_	antibacterial activity	[77]
**2**	3*b*-acetoxyl,2,14,22-trihydroxy,19-hydroxymethyl,9*a*,5*b*,14*b*-card-20(22)enolide	C_25_H_36_O_8_	antibacterial activity	[78]
**3**	1,2,3,4-tetrahydro-6-hydroxy1-5-pyrimidinecarbox-aldehyde	C_5_H_8_N_2_O_2_	antibacterial activity	[78]
**4**	(–)-7*S*,8*S*,9*R*,13*R*,14*S*,17*R*,20*S*(22*E*)-3-oxocholesta-4,22(23)-dien-25-ol	C_27_H_43_O_2_	antibacterial activity	[86]
**5**	(–)-7*S*,8*S*,9*R*,13*R*,14*S*,17*R*,20*S*-(22*E*)-3*b*-acetate,5(6)-22*E*-(23)-cholestadien	C_29_H_47_O_2_	antibacterial activity	[86]
**6**	(–)-1*b*-methyl-6*R*-hydroxyl-bicyclo-[0,2,4]-hex-1-ene-4*a-S*-(2¢-methyl)-isooctanoic	C_15_H_25_O_3_	antibacterial activity	[86]
**7**	trigonelline	C_7_H_8_NO_2_	anti-inflammatory activity	[87]
**8**	*N*-(4-guanidinobutyl)-4-hydroxybenzamide	C_12_H_18_N_4_O_2_	inhibitory activity against both acetylcholinesterase and butyrylcholinesterase	[87]
**9**	*N*-(4-guanidinobutyl)picolinamide	C_11_H_17_N_5_O	inhibitory activity against cholinesterase	[87]
**10**	3-methylbuthyl hydrodisulfide	C_5_H_13_S_2_	-	[87]
**11**	harmanyl *b*-*D*-glucopyranoside	C_18_H_20_N_2_O_6_	inhibitory activity against *α*-glucosidase	[88]
**12**	cholesterol	C_27_H_46_O	composition of cell membrane	[89]
**13**	pregn-5-ene-3,20-diol	C_21_H_34_O_2_	-	[89]
**14**	cholesteryl palmitate	C_43_H_76_O_2_	-	[89]
**15**	cholest-4-en-3-one	C_27_H_44_O	-	[90]
**16**	1-stearyl-glyc-ero-3-phosphocholine	C_26_H_54_NO_7_P	-	[90]

### 2.7. Identification and Application of Active Components

In recent years, researchers have identified and analyzed the chemical components of CS using various advanced sequencing technologies, leading to the discovery of peptides and proteins with therapeutic potential. For instance, proteomics based on MS and MS/MS can be efficient methods for the rapid analysis of toxins [91].

**Figure 8:**
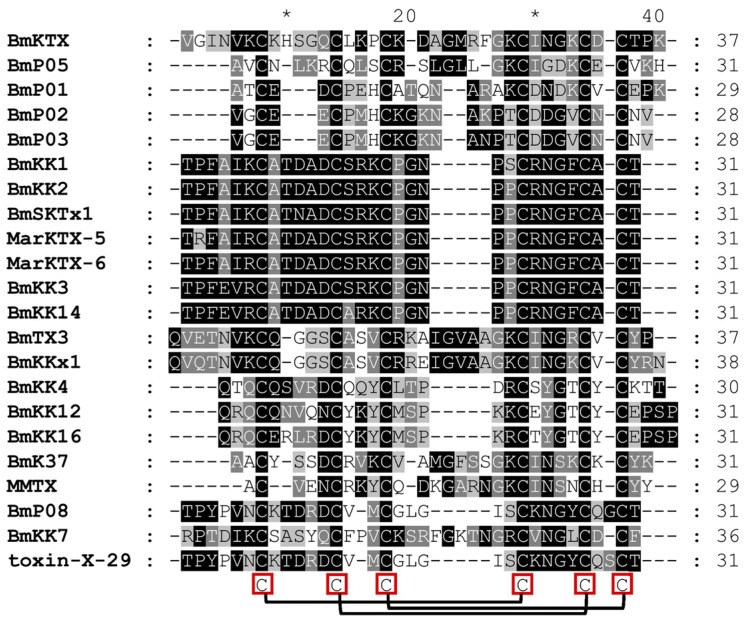
Sequence alignment of the 22 identified full-length scorpion toxins. Similarities in sequences are indicated with shading. The six conserved cysteine residues in all toxins, capable of forming three disulfide bonds, are illustrated by the red box with connecting lines [92] (reproduced with permission from Yingliang Wu, Journal of Proteomics; published by Elsevier, 2019).

Traditional Chinese medicinal materials are usually processed to reduce toxic side effects before clinical application. However, whether the active components are altered during this process remains a critical question. Yang et al. [92] conducted a comprehensive mass-spectrometry-based proteomic characterization and identified 22 full-length potassium-channel-inhibiting neurotoxins, which all contained six cysteine residues capable of forming three conserved disulfide bonds (Figure 8). These neurotoxins were found to have therapeutic potential in treating Kv1.3-channel-related immune diseases [92]. The identification of these 22 full-length neurotoxins indicated that the bioactivity of chemical components was still maintained after thermal processing. This finding supports the feasibility of thermal processing before the therapeutic applications of CS.

Qin et al. [93] used proteomic methods to identify a novel peptide, BmK86-P1, which is highly potent and selective for the Kv1.2 channel in thermally processed scorpion. Meanwhile, circular dichroism (CD) spectroscopy analysis revealed that the secondary structures of the BmK86-P1 remained thermally stable within 20–95 °C. The thermostability of BmK86-P1 indicates the potential use of thermally processed scorpions in treating Kv1.2 channel-related diseases [93]. This finding not only expands the therapeutic possibilities of CS but also ensures the efficacy and safety of its clinical applications.

In addition, proteomics can also be used for the quality analysis of CS. The current legal quality standard for CS is the 2020 edition of the Chinese Pharmacopoeia, covering properties, microscopic identification, moisture, ash, and aflatoxin inspection items. However, it lacks thin-layer chromatography identification and content determination, providing only a partial reflection of the quality of CS [7]. Liang et al. [94] selected β-actin and its characteristic peptide segments, which are suitable for content determination through using proteomics technology, and established a method to determine the contents of β-actin and its characteristic peptide segments based on UPLC-MS. This provides an effective method for the quality control of CS.

## 3. Discussion

CS, a traditional Chinese medicine, exhibits significant clinical value despite its highly complex chemical composition. Scorpion venom peptides, the most important components, exhibit a wide variety of types, structures, and functions [2]. These peptides exert different pharmacological activities by specifically binding to various ion channels. Even when targeting the same ion channel, they display various pharmacological activities due to differences in peptide structure, affinity, or signaling pathways. For example, peptides targeting sodium ion channels can exhibit analgesic, antiepileptic, and antitumor effects [24,25]. Therefore, the scorpion venom peptides in CS hold considerable research value.

The interaction between various chemical components in CS and ion channels may exhibit synergistic effects, providing more complex and diverse mechanisms for its pharmacological actions. For instance, cardiotonic steroids primarily bind to Na^+^-K^+^-ATPase, inhibiting its activity and affecting the transmembrane transport of sodium and potassium ions, thereby regulating the ionic balance inside and outside cells [74,75]. Additionally, CS contains scorpion venom peptides that specifically target sodium and potassium ion channels [12,22]. These components may affect ion channel function through different mechanisms, thus exerting synergistic effects in physiological processes such as nerve signal transmission, muscle contraction, and cardiac rhythm. This interaction mechanism holds promise for the development of new drugs.

This review provides a detailed examination of the effects of a traditional Chinese medicine, CS and its active components, on ion channels. It explores its traditional medicinal value and potential as a source for novel drugs. The chemical constituents of CS are complex, exhibiting structural and functional diversity, which allows them to bind specifically with various ion channels and exert diverse pharmacological effects. However, research into the material basis of the efficacy of CS remains insufficient, particularly regarding the pharmacological mechanisms of small-molecule compounds. Future studies should focus on elucidating the structure and function of these compounds, potentially starting with their interactions with ion channels. In summary, as a traditional Chinese medicine, CS possesses a wealth of pharmacologically active ingredients and broad application prospects. Further research is needed to deepen our understanding of its material basis, develop new drugs, and establish improved quality control standards to better serve human health.

## Data Availability

Data sharing is not applicable to this article as no new data were created or analyzed in this study.
